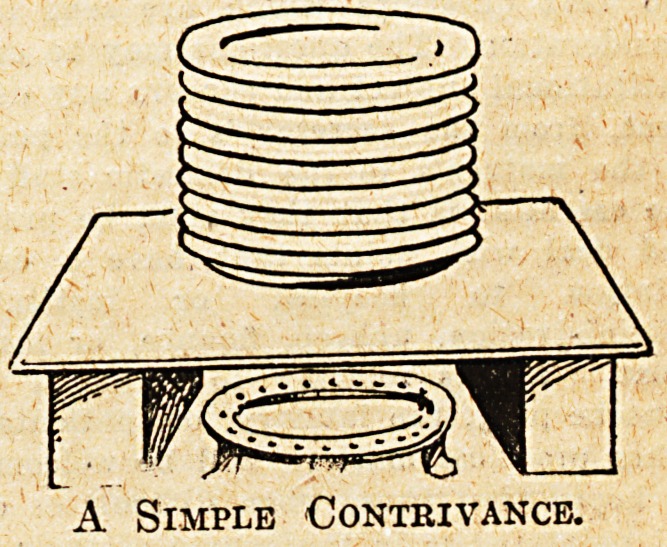# The Institutional Worker

**Published:** 1918-04-06

**Authors:** 


### THE INSTITUTIONAL WORKER

**Being a Special Supplement to "The Hospital"**

### OUR BUREAU OF INFORMATION.

### Rules for Correspondents.

1. Every letter must be accompanied by the coupon to be cut from the back cover (inside page) of The Hospital, current issue, and must contain the name and address of the correspondent with pseudonym for publication if desired. Replies by post cannot be given save under exceptional circumstances at the Editor's discretion.

2. Letters from Approved Homes in reply to special needs published in the Bureau should state terms and full particulars, and be sent prepaid under cover to the Editor of the Bureau with name written across coupon for identification.

3. Proprietors of Homes which have not yet been entered on the List of Approved Homes, but have spare accommodation likely to suit special needs, are invited to write for an application form for registration. The fee for registration, which includes two announcements of the Home in the Bureau and other privileges, is 10s.

4. All communications to be addressed to the Editor of The Hospital, 28 Southampton Street, Strand, London, W.C. 2, and marked " Bureau of Information."

### INSTITUTION FACTS AND FIGURES.

### Hot Dinner-Plates.

#### Answer to February Question.

**The question was :**

**A hospital has four wards, each containing twenty beds. The dinners are served from one inadequate general kitchen and there is no arrangement for heating the plates. Each ward has a very tiny kitchen, which holds a small gas-ring. How can the plates be best heated ?**

#### By J. R. Y.

It is preferable that the plates for each ward should be warmed by means of an apparatus in its own kitchen rather than at the general kitchen, so that the meals may be quickly served to the patients while the plates are quite warm.

If there is a fair-sized coal range it may be sufficient for the purpose, but it is generally more convenient to have a plate-warming oven of suitable dimensions to accommodate the number of plates required.

Since gas is already installed in the kitchens this may be used for heating the ovens, which latter may be obtained from any firm specialising in the manufacture of cooking apparatus, or they may be made by the hospital engineer.

We may assume that two dinner-plates are required for each patient, so that accommodation will be required to heat forty plates, each, say, 11 in. diameter.

The apparatus may consist of a sheet-iron box or oven, open at the bottom, provided at the front with hinged door with catch or bolt, having two grids, each to hold a pile of twenty plates, and warmed by means of four ordinary flat-flame gas-burners fixed horizontally along the lower portion of the oven, the burners being arranged so that the flames spread out in a horizontal position, thus diffusing the heat evenly.

The inside dimensions of the oven will be 13 in. wide, 12 in. deep, and 24 in. high, the door being the full width of the front and extending from the top to within 5 in. of the base. There will be a space 2 in. high along the lower portion of the front to admit air to the burners, and three $1\frac{1}{2}$-in. holes along each end near to the top to allow the products of combustion to escape.

The burners will be $2\frac{1}{2}$ in. from the base of the oven, the top of the lower grid being $2\frac{1}{2}$ in. above this, and the upper one midway to the top of the oven. This will allow a clear space of about 8 in. between the shelves, which will be sufficient for the piles

A Plate Warming Oven.

of plates to be placed in or removed from the oven.

The gas-ring burner already fixed in each kitchen may now be placed on the top of the oven and the whole mounted, for convenience, on a box or stand about 10 in. high. The space occupied will thus be little more than that at present required for the burner itself.

If the stand is of wood it will be advisable to cover it with sheet iron, or, better still, with sheet iron with asbestos millboard underneath as a precaution against fire.

An improvised apparatus can be made in the following manner by utilising the existing gas-ring : —

Procure a tin box of suitable size to take a pile of forty plates, and stand it on the gas-ring with its open side to the front, which should have a hinged door or loose lid. The box must be secured, say, to the wall, to keep it steady. A number of holes punched round the outer portion of the bottom will allow the heated gases to enter and pass up among the plates, while others punched near the top will allow these products of combustion to escape. Place a circular piece of asbestos or small iron stand on the bottom of the box on which to stand the plates, so that the lower ones will not be overheated.

#### By E. M. C.

In answer to the question *re* "Hot Dinner-Plates," I suggest that if pouring boiling water over them is impracticable, the only other alternative would be to place two bricks, one on each side of the gas-ring, and a piece

A Simple Contrivance.

of sheet iron (a baking-sheet would do) to cover the whole. The plates could then be placed on the sheet, and when the gas was lit the iron would get thoroughly hot and the plates, by changing them from the top to the bottom, would get perfectly hot. This plan would also be useful for keeping food hot.

**The Editor will be glad to receive correspondence and to consider contributions upon all subjects relating to institutional work which affect the welfare of institutional workers.**

### APRIL QUESTION.

### A Register of Nurses' Ward Work

**How should a book be ruled to give at a glance a record of nurses' different duties in male, female, and children's wards, and time spent at classes, etc.? The hospital is a training-school, and nurses take day and night duty. There are three floors for male patients, two for female patients, and a children's block.**

#### RULES.

The following rules must be observed:—

1. Contributions must be written on one side of the paper. Brevity and terseness are desirable features. The MSS. must bear the name and address of the sender and be accompanied by coupon to be cut from the back cover (inside page) of the current issue of THE HOSPITAL. A pseudonym must be chosen if the name is not to be published.

2. Contributions must be addressed to the Editor of THE HOSPITAL, Institutional Worker Supplement, 28 & 29 Southampton Street, Strand, London, W.C. 2, should reach him before the end of the current month, and be marked in the left-hand corner "Facts and Figures."

**A minimum payment of Five Shillings will be made for each published answer.**

### QUESTIONS INVITED.

IN connection with our Question Box, we offer every month five shillings each for the two best questions which are sent in for consideration. The questions may be on any institutional subject and concern any department, from the secretary's to the porter's, or the matron's to the domestic staff; the one essential is that they must be practical and deal with points that have a definite relation to hospital or institutional administration, upkeep, finance, or management. Points relating to artificers' work, housekeeping, the laundry, out-patients, and other practical matters will be welcome. It is hoped that thus, when difficult or doubtful points arise in the course of their work, institutional workers will be encouraged to put them in the form of a question and send them to the Editor, THE HOSPITAL Bureau of Information, 28 & 29 Southampton Street, Strand, W.C. 2, marked "Question Box."

The best questions will be published in due course and our readers will be given the opportunity of answering them. Thus all institutional workers may be able to cooperate with the view of helping the work and smoothing the difficulties of each other. In short, we want the Question Box of the *Institutional Worker* to be freely used by every worker habitually.

### ENQUIRIES AND ANSWERS.

### *THE SICK AND IN NEED.*

### Consumption Sanatorium.

You might possibly be accepted as a patient at the Newcastle-upon-Tyne and Northumberland Sanatorium, Barrasford, Northumberland. Each patient is provided with a separate bedroom. Patients residing out of the districts—*i.e.*, Newcastle-upon-Tyne and Northumberland—are required to pay £2 10s. per week, or £30 in one sum in advance for three calendar months. These charges may, of course, have been revised owing to increased cost of living. Both male and female patients are taken.—CORNFORTH.

### Private Asylum near London.

As you do not state what fees you are prepared to pay it is somewhat difficult to advise you. One of the following institutions, however, may be suitable for the case in question:—Chiswick House, Chiswick; this stands in sixty-three acres of grounds, and is six miles by road from Hyde Park Corner. Write to the Medical Superintendent for full particulars, Northumberland House, Green Lanes, Finsbury Park, N.; it is highly situated in six acres of grounds, facing Finsbury Park, and has private villas in suites of rooms. Voluntary boarders are taken at both institutions.—REST.

### *EMPLOYMENT AND TRAINING.*

### Assistant Secretaryship Required in Large General Hospital.

WE regret that we cannot do more than advise you to watch the advertisement columns in THE HOSPITAL and nursing journals, also the leading daily papers, and reply to suitable advertisements as they appear. You might also make your needs known by advertising.—E. R. W.

### Clerkship in Hospital Office.

SEE reply to E. R. W. in these columns. You do not require to study medicine for a position such as you require. A coupon should accompany each inquiry, with full name and address.—L. B. I.

### Midwifery and Massage Training at Ipswich.

PUPILS are trained in midwifery and massage at the Ipswich Nurses' Home and Training-school for Midwifery and Massage, 7 Lower Brook Street, Ipswich. The fees for midwifery, including board and lodging, are £16 16s. for three months, £21 for four months. Bags, laundry, and examination fees are extra. Pupils are also prepared for the examination of the Incorporated Society of Trained Masseuses, the terms for which may be obtained upon application to the Matron.—ISSA.

### Post in London Hospital.

YOU should experience no difficulty in obtaining a post in a London hospital, having regard to the fact that you hold a three years' certificate of a recognised training-school. Owing to the war there is a dearth of nurses for civil patients. Watch the advertisements which appear in the nursing journals each week.—GWLADYS.

### Training at Tunbridge Wells.

THE hospital you mention has under one hundred beds. Candidates are received for three years' training at the age of twenty-one to thirty. Training is given in all branches of general hospital nursing, and a course of massage lessons is given. Nurses also gain experience in *x*-ray and electrical work. The hospital contains twenty-six beds for wounded.—BELINDA.

### *WAR MATTERS.*

### British Dogs' Wool Association.

AN object which will undoubtedly appeal to all patriotic dog-owners has just been promoted under the auspices of the British Red Cross Society. An Association, named the British Dogs' Wool Association, has been formed with a view to providing the central workrooms of the British Red Cross Society with wool obtained from the combings of long-haired dogs such as Chows, Pekingese, poodles, Bergecs d'Alsace, and sheep-dogs, for the benefit of the sick and wounded. Wool spun from combings of these dogs has been submitted to wool experts, who are highly in favour of its use. Owners are asked to preserve the combings from their dogs, and any inquiries may be addressed to the Hon. Sec., Miss L. C. Smythe, British Red Cross Society Central Workrooms, Royal Academy, Piccadilly, W. 1

### Nurses' Memorial Service at St. Paul's.

THE service you refer to will take place on April 10, at 2.30 P.M., in St. Paul's Cathedral. Nurses in uniform and relatives of those in the Roll of Honour will have spaces reserved for them in the cathedral. You must apply to the Rev. A. Lombardini, Church of St. Elizabeth, Kensington, W. 8.—NURSE E.

### *THE HOUSEKEEPERS' DEPARTMENT.*

### Vegetables as a Meal.

NO, vegetables do not provide a complete meal. They are deficient in fat, but of course this may be made up by the use of butter, margarine, dripping, or oil. Also cheese added increases the percentage of body-building and tissue-repairing material. Vegetables have a high percentage of mineral ingredients which are very necessary to life; they contain also a large amount of pure soft water—W. M. C.

## Figures and Tables

**Figure f1:**
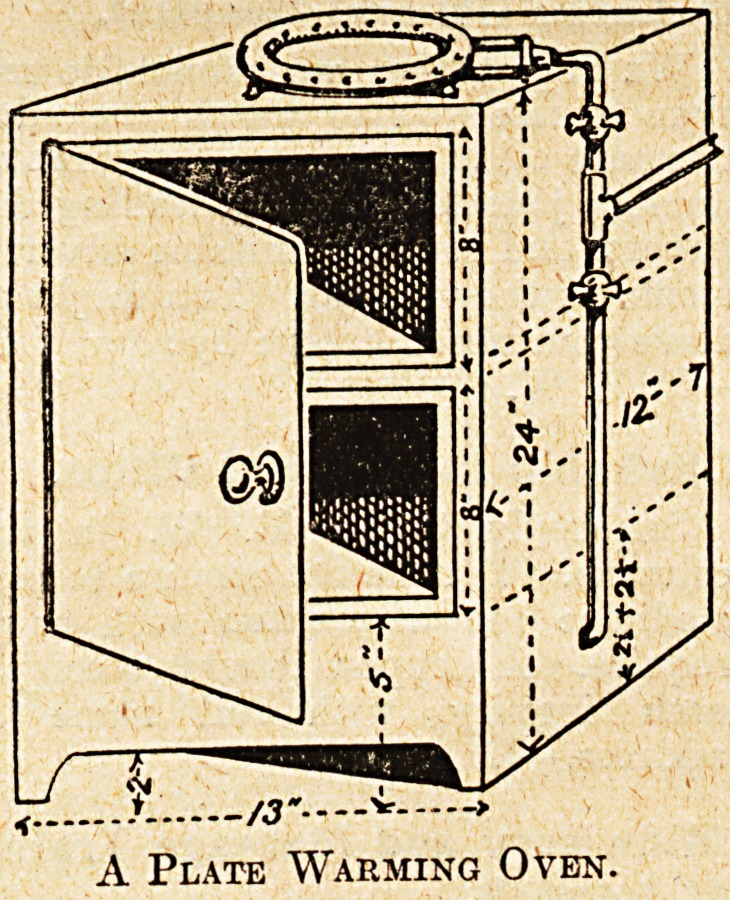


**Figure f2:**